# Inverse planning optimization with lead block effectively suppresses dose to the mandible in high-dose-rate brachytherapy for tongue cancer

**DOI:** 10.1007/s11604-023-01451-w

**Published:** 2023-06-05

**Authors:** Yuichi Akino, Hiroya Shiomi, Tomomi Tsujimoto, Noriaki Hamatani, Takero Hirata, Michio Oda, Ami Takeshita, Hiroaki Shimamoto, Kazuhiko Ogawa, Shumei Murakami

**Affiliations:** 1 Department of Radiation Oncology, Osaka University Graduate School of Medicine, 2-2, Yamadaoka, Suita, Osaka 565-0871 Japan; 2 Department of Oral and Maxillofacial Radiology, Osaka University Graduate School of Dentistry, Suita, Osaka Japan; 3 Department of Medical Physics, Osaka Heavy-Ion Therapy Center, Osaka, Japan; 4 Department of Medical Technology, Osaka University Hospital, Suita, Osaka Japan

**Keywords:** Interstitial brachytherapy, Tongue cancer, Lead block, Optimization

## Abstract

**Purpose:**

In this study, we developed in-house software to evaluate the effect of the lead block (LB)-inserted spacer on the mandibular dose in interstitial brachytherapy (ISBT) for tongue cancer. In addition, an inverse planning algorithm for LB attenuation was developed, and its performance in mandibular dose reduction was evaluated.

**Methods:**

Treatment plans of 30 patients with tongue cancer treated with ISBT were evaluated. The prescribed dose was 54 Gy/9 fractions. An in-house software was developed to calculate the dose distribution based on the American Association of Physicists in Medicine (AAPM) Task Group No.43 (TG-43) formalism. The mandibular dose was calculated with consideration of the LB attenuation. The attenuation coefficient of the lead was computed using the PHITS Monte Carlo simulation. The software further optimized the treatment plans using an attraction–repulsion model (ARM) to account for the LB attenuation.

**Results:**

Compared to the calculation in water, the D_2 cc_ of the mandible changed by − 2.4 ± 2.3 Gy (range, − 8.6 to − 0.1 Gy) when the LB attenuation was considered. The ARM optimization with consideration of the LB resulted in a − 2.4 ± 2.4 Gy (range, − 8.2 to 0.0 Gy) change in mandibular D_2 cc_.

**Conclusions:**

This study enabled the evaluation of the dose distribution with consideration of the LB attenuation. The ARM optimization with lead attenuation further reduced the mandibular dose.

## Introduction

High-dose-rate interstitial brachytherapy (HDR-ISBT) enables highly conformal irradiation of a target while reducing the dose delivered to surrounding healthy tissue using low-energy γ rays. Several studies have reported good clinical outcomes of HDR-ISBT for the treatment of tongue cancer [1–3]. Because tongue cancer typically arises at the lateral border of the tongue, the nearby gingiva and mandible are often irradiated with high doses. These sites can be subject to serious complications, including osteoradionecrosis and osteomyelitis [4, 5]. In the treatment of tongue cancer, spacers can be used to increase the distance between the tumor and the lower jaw, thereby reducing the dose delivered to the mandible [6]. In our institution, a lead block (LB) is inserted into the spacer to further reduce the mandible dose using the shielding effect of γ ray absorption by high-atomic-number lead [7].

Currently, many institutions calculate the dose distribution of brachytherapy treatment plans based on the American Association of Physicists in Medicine (AAPM) Task Group No. 43 (TG-43) update 1 formalism [8–10]. Because the TG-43 formalism calculates the dose in a water-equivalent material, the calculated dose distribution cannot account for the attenuation of the γ rays by the lead block. In recent years, model-based dose calculation algorithms have been incorporated into commercial treatment planning systems (TPS) [11, 12]. In addition, inverse planning optimization algorithms for brachytherapy are available [13]. However, no technique has been reported to optimize the dose distribution by inverse planning, taking into account the LB attenuation.

In this study, we developed in-house software to evaluate the effect of the LB-inserted spacers on mandibular dose in ISBT for tongue cancer. In addition, an inverse planning algorithm that accounts for LB attenuation was developed and its performance in mandibular dose reduction was evaluated.

## Methods

### Patients

**Fig. 1:**
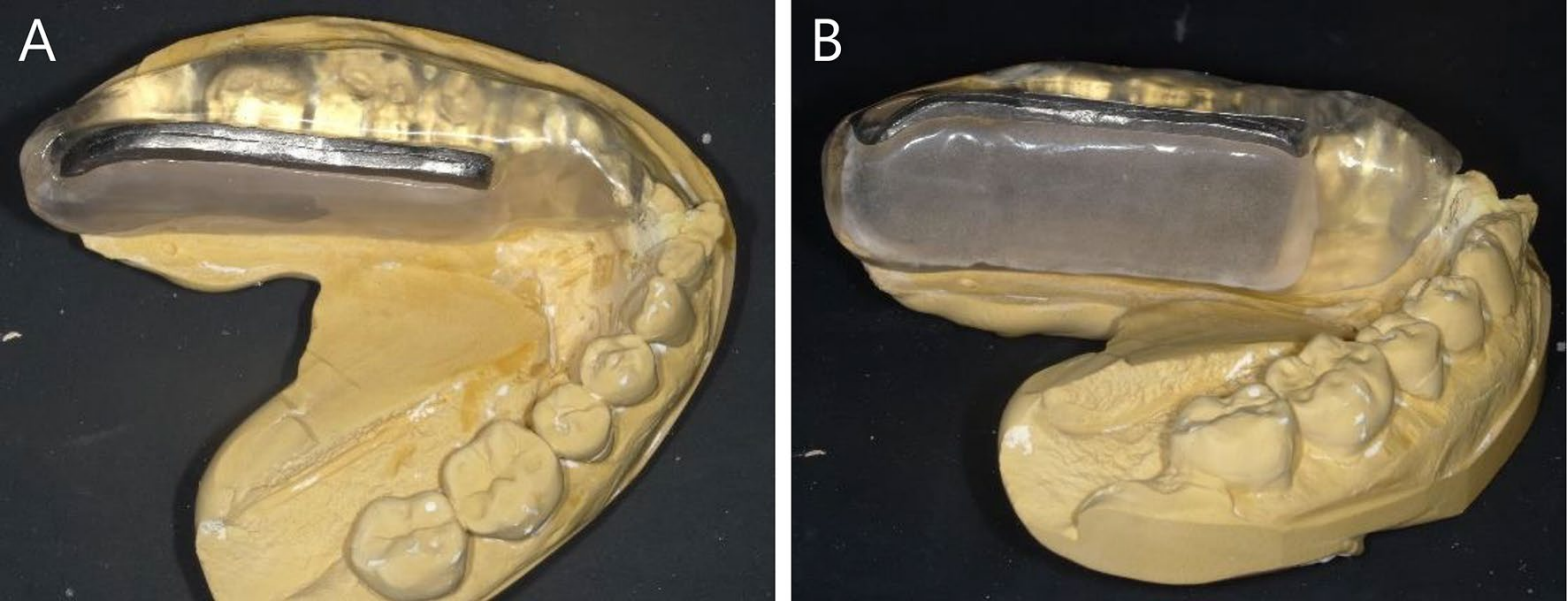
Photographs of the spacer containing the lead block

Under institutional review board approval, 30 patients with tongue cancer treated with ISBT between September 2018 and August 2021 were evaluated. Planning target volume (PTV) was defined as the same as gross tumor volume (GTV). The mean ± standard deviation (SD) of the PTV was 11.7 ± 7.9 cm^3^ (range, 2.7–28.6 cm^3^). Needles were inserted under the jaw into the oral cavity and replaced with flexible plastic catheters. The number of applicators used was 5.9 ± 2.4 (range, 3–14). All patients were treated with a spacer made of resin inserted between the mandible and tongue. A 3-mm-thick LB was inserted into the spacer (Fig. 1). Details of the spacer with the lead block are described elsewhere [7]. The LB was removed during image acquisition of the planning CT to avoid the effects of metal artifacts. The Oncentra Brachy (Elekta, Stockholm, Sweden) TPS was used for treatment planning. The prescribed dose was 54 Gy/9 fractions/5 days [14]. Graphical optimization was used to improve PTV coverage and reduce the mandibular dose.

### Dose calculation

The dose distributions of the clinical treatment plans were calculated using the AAPM TG-43 update 1 formalism [8, 9]. In this study, dose distributions with and without LB attenuation were calculated using a ShioRIS Brachy (SRB), which was developed in-house as a derivative of the commercial ShioRIS 2.0 software (RADLab Inc., Osaka, Japan). For dose calculation in the SRB, a three-dimensional (3D) dose distribution of a single dwell point was calculated by the Oncentra Brachy TPS and was exported as DICOM RT-Dose file format with a spatial resolution of 1 mm. To calculate the dose at a voxel, the position relative to each dwell point was calculated and the dose at the same relative position from the single dwell point was corrected by the dwell time and air kerma strength of the clinical plan. The calculation grid size of patient data was 1 × 1 mm in transverse plane, while the longitudinal resolution was equal to the slice thickness of the CT images. To evaluate the accuracy of the SRB dose calculation in a water-equivalent medium (SRB_w_), the dose distributions of the clinical plans calculated with the SRB were compared to those calculated with the Oncentra Brachy.

### Lead block attenuation

**Fig. 2:**
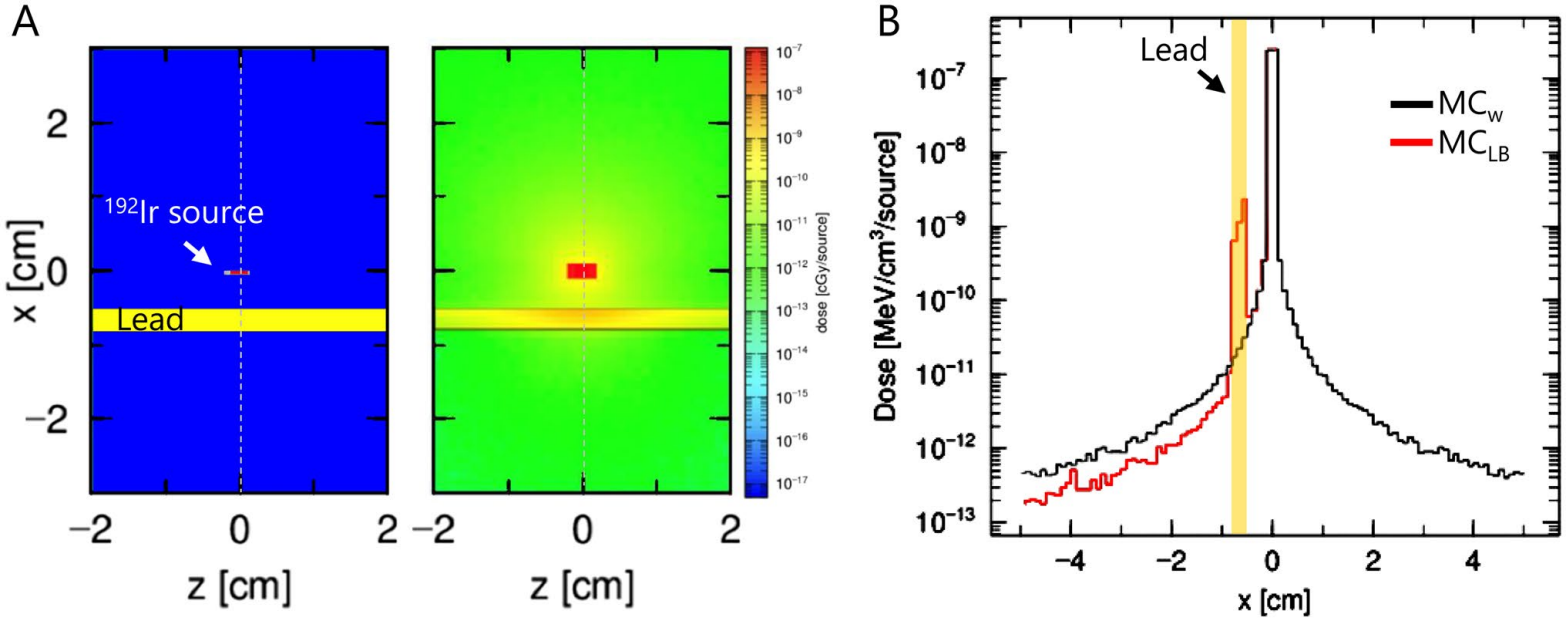
**A** Geometry (left panel) and dose distribution (left panel) of the ^192^Ir source calculated by the PHITS Monte Carlo code. **B** Dose profile of the vertical axis passing through the ^192^Ir source. MC_w_, Monte Carlo calculation in water; MC_LB_, Monte Carlo calculation in water with the lead block

To account for LB attenuation in the 3D dose calculations, the microSelectron HDR v2 revision (mHDR-v2r) ^192^Ir source [15] was modeled with a PHITS Monte Carlo (MC) code [16], and the distribution of radiation emitted by the source in water was simulated by calculating 6.6 × E + 09 particles. The self-absorption of the stainless-steel capsule and source was considered for dose calculation, but the wire was not considered. A 3-mm-thick LB was placed 5 mm from the ^192^Ir source, and the attenuation coefficient of LB was calculated by the dose profile downstream of the block (Fig. 2A). The calculated attenuation coefficient of LB was used for dose calculation by the SRB with consideration of the LB attenuation (SRB_LB_). When calculating the total dose of a voxel, the dose delivered from each dwell position was separately calculated. Then, the thickness of the lead block along the path between the dwell position and the voxel was measured with the block contour data of the DICOM RT-Structure file. The attenuation for each dwell position was calculated by the block thickness and the attenuation coefficient, and the value was multiplied to the dose delivered from the dwell position.

### Optimization of treatment plans with consideration of the lead block

**Fig. 3:**
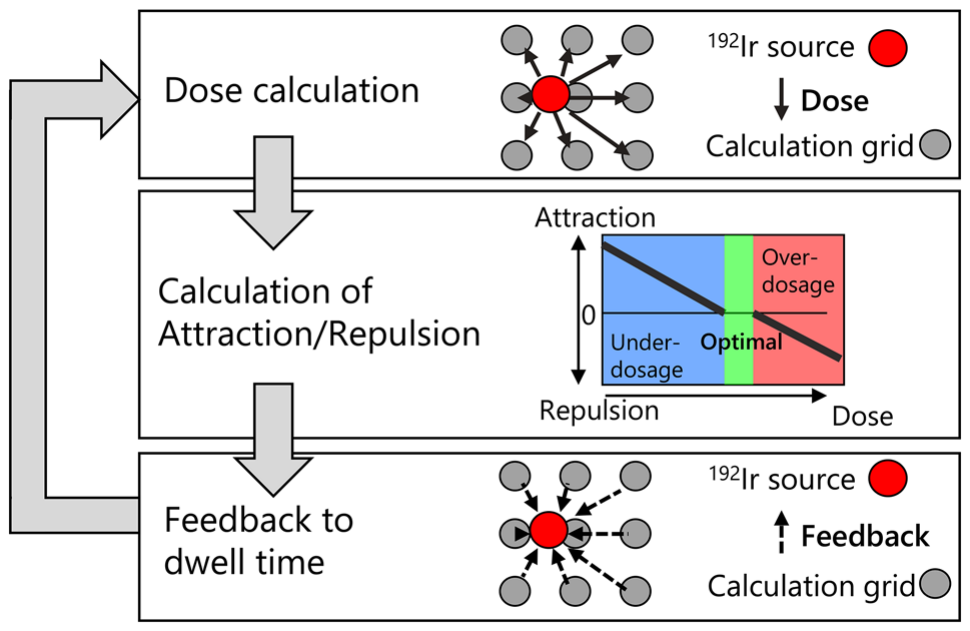
Flowchart of the optimization of treatment plans using the attraction–repulsion model

To further improve dose distribution with consideration of LB attenuation, an optimization algorithm using the attraction–repulsion model (ARM) was developed. The ARM is based on Gauss’ law regarding the distribution of charged particles in an electric field. The charged particles representing the dwell time of an HDR iridium source at each dwell position are affected by the Coulomb forces from the electric field [17]. Figure 3 shows a flow chart of the ARM optimization process. For example, the calculation grid in Fig. 3 represents the voxels within the PTV. For each voxel, the dose delivered from each dwell position was calculated, and the accumulated total dose and the contribution factor for each dwell position are calculated (Dose calculation). If the total dose in the voxel exceeds the upper limit of the dose constraint, the voxel generates repulsion force that reduces the dwell time. If the total dose is less than the lower limit, the point generates an attraction force to increase the dwell time (Calculation of Attraction/Repulsion). According to the contribution factor, the dwell time of each source dwell position is regulated by the voxel (Feedback to dwell time). The PTV and mandible were segmented into voxels with resolution of 1 × 1 mm in transverse plane, while the longitudinal resolution was equal to the slice thickness of the CT images. All voxels within the PTV and mandible worked as optimization points.

### Evaluations and statistical analyses

Clinical treatment plans were optimized using a graphical optimization function of the Oncentra Brachy. There was no institutional protocol for dose normalization after graphical optimization. For evaluation of the LB attenuation, the 3D dose distributions of the clinical plans were re-calculated by SRB_w_ and SRB_LB_ with original source strength and dwell time. In contrast, for evaluation of the effectiveness of the ARM optimization, both the clinical plans calculated with the SRB_w_ and the ARM-optimized plan (SRB_ARM_) were rescaled to the PTV dose covering 95% (PTV D_95%_) = 100% of the prescribed dose.

Doses delivered to 1 cm^3^, 2 cm^3^ and 5 cm^3^ (D_1 cc_, D_2 cc_, and D_5 cc_, respectively) of the mandible were evaluated. Differences in the dose-volume histogram (DVH) parameters between dose calculation methods were compared using the paired *t* test. Statistical significance was defined as a *p* value < 0.05.

## Results

### Dose calculation with consideration of the lead block

The dose calculation accuracy of the SRB was evaluated by comparing the dose distributions of the clinical plans with those calculated with the Oncentra Brachy. For 5,343 voxels receiving doses between 2 and 10 Gy, 99.89% of the voxels showed good agreement within 1%.

Figure 2B shows the dose profile along the dashed line in Fig. 2A. The dose profile calculated with MC with consideration of the LB attenuation (MC_LB_) decreased significantly downstream of the LB compared to the MC calculation in water (MC_w_). A quadratic fitting function of the dose profile downstream of the LB was calculated for smoothing. The ratio of MC_LB_ and MC_w_ was 0.470 at 5 mm downstream of the LB. Because the LB was 3 mm thick, the half-value layer was 2.76 mm.

**Fig. 4:**
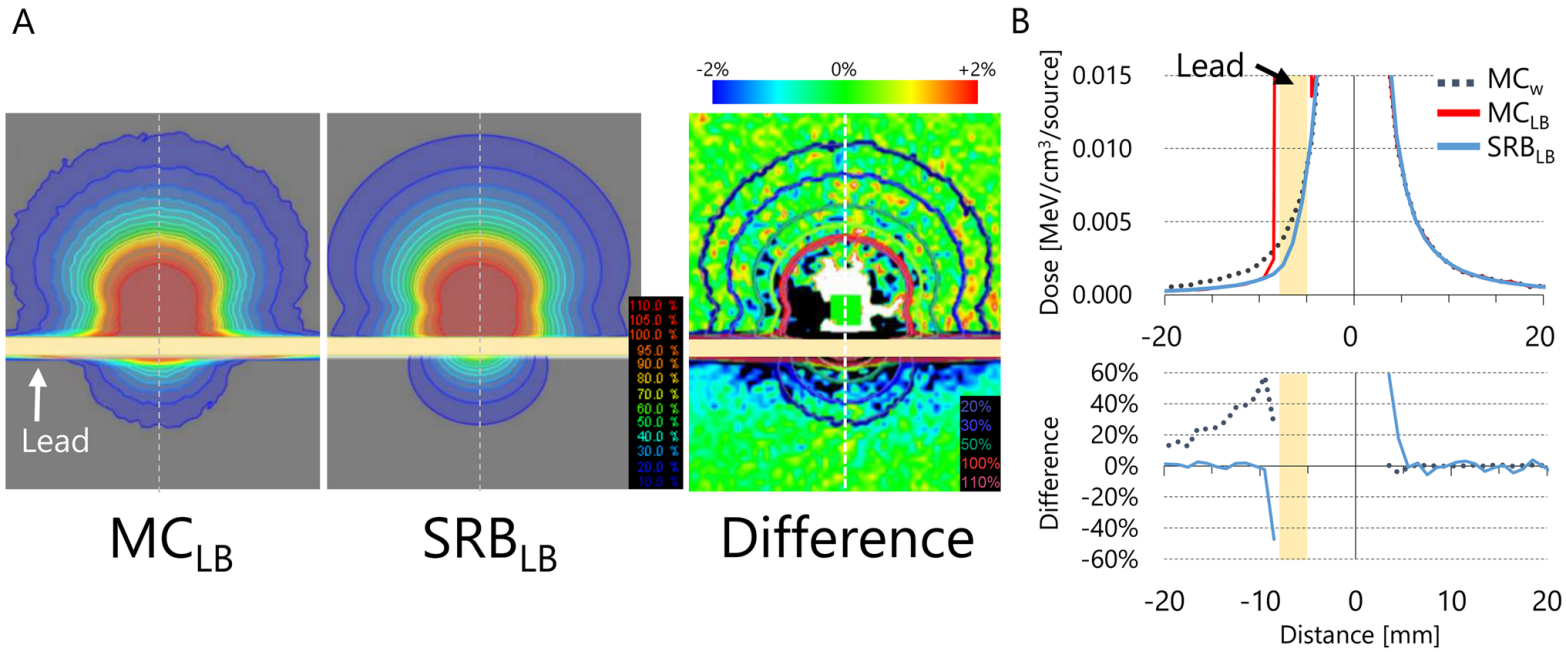
**A** Dose distribution of the ^192^Ir source calculated with MC_LB_ and SRB_LB_. **B** Top panel: dose profile of the vertical axis passing through the ^192^Ir source (shown as dashed lines in Figure **A**). Bottom panel: the difference of MC_w_ and SRB_LB_ from MC_LB_. MC_w_, Monte Carlo calculation in water; MC_LB_, Monte Carlo calculation in water with the lead block; SRB_LB_, calculation with the ShioRIS Brachy with consideration of the lead block attenuation

Figure 4A shows the MC_LB_- and SRB_LB_-calculated dose distribution of the single dwell point considering the 3-mm-thick LB placed 5 mm from the source. The dose difference between MC_LB_ and SRB_LB_ was also shown. Except for the region very close to the source and LB, the MC_LB_ and SRB_LB_ showed good agreement within 2%. MC_LB_ showed clear dose increase near the lead block due to the backscatter from the lead block. SRB_LB_ did not show such dose increase, because only the attenuation coefficient was considered. In Fig. 4B, the top panel shows the vertical dose profile along the dashed line shown in Fig. 4A. The bottom panel of Fig. 4B shows the difference of MC_w_ and SRB_LB_ from MC_LB_. The dose within LB showed large difference, because the SRB did not consider the energy deposition according to the material. The SRB_LB_ showed good agreement with the MC_LB_ downstream the LB, indicating that the SRB_LB_ enables a more realistic evaluation of the mandibular dose.

**Fig. 5:**
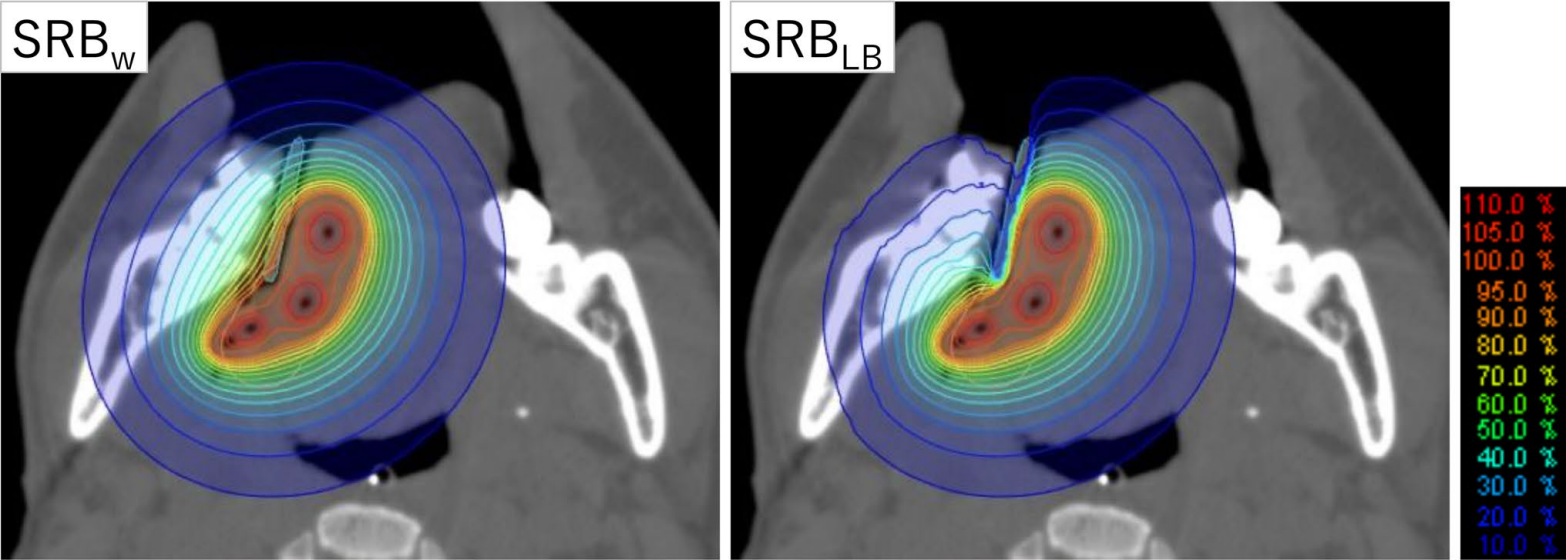
Dose distribution of an example clinical plan calculated with ShioRIS Brachy in water (SRB_w_) and that with consideration of lead block attenuation (SRB_LB_)

**Fig. 6:**
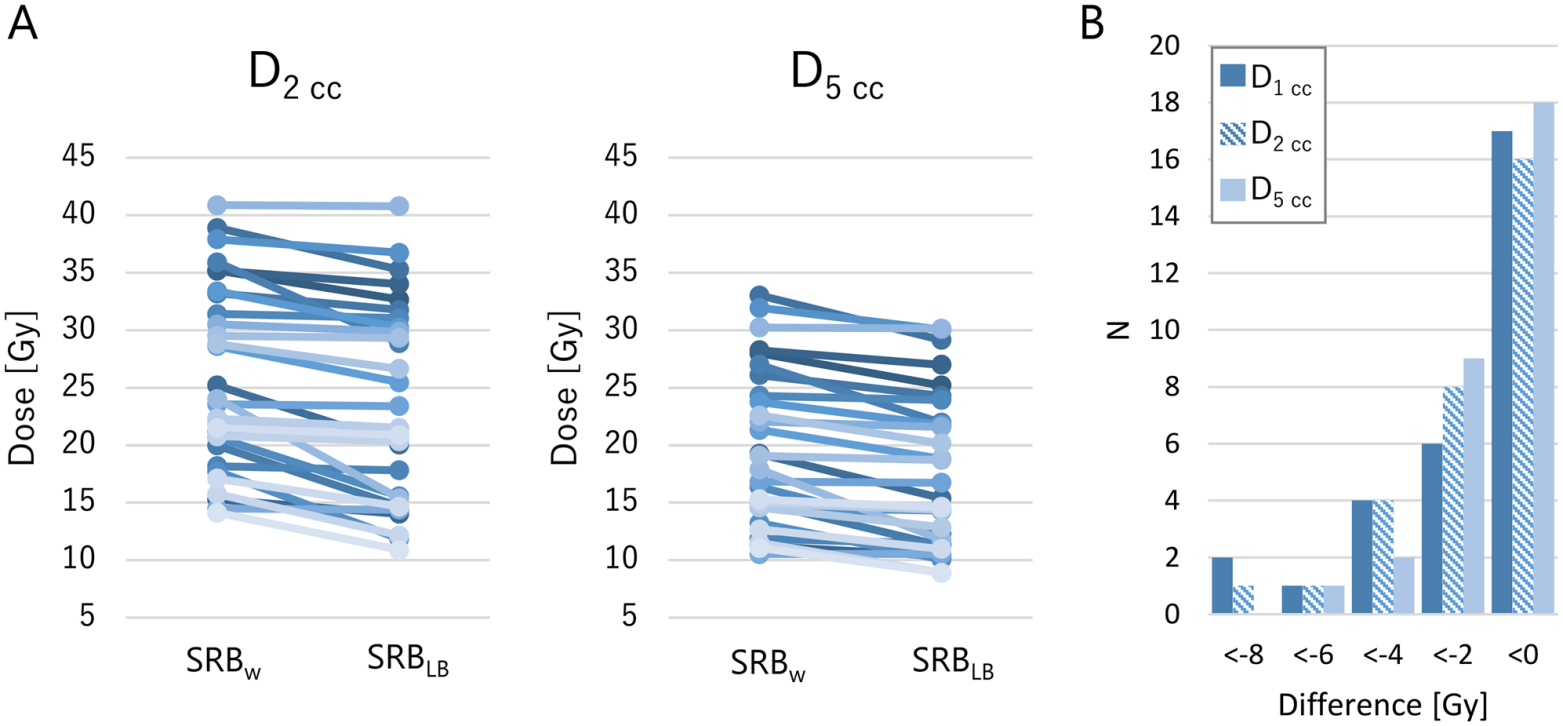
**A** Dose delivered to 2 cm^3^ (D_2 cc_) and to 5 cm^3^ (D_5 cc_) of mandible calculated by ShioRIS Brachy. in water (SRB_w_) and that with consideration of lead block attenuation (SRB_LB_). **B** Number of cases plotted against the differences of the D_1 cc_, D_2 cc_, and D_5 cc_ between SRB_w_ and SRB_LB_

Figure 5 shows the dose distributions of an example clinical plan calculated by SRB_w_ and SRB_LB_. The dose behind the block decreased significantly. Figure 6A shows the D_2 cc_ and D_5 cc_ of each patient. Not all, but some cases showed that the DVH parameters decreased by LB. In Fig. 6B, the number of cases is plotted against the differences in DVH parameters between SRB_w_ and SRB_LB_. Mean ± SD of the changes in the DVH parameters are listed in Table 1. D_2 cc_ decreased by an average of 2.4 Gy with LB. The relative difference in D_2 cc_ was − 10.2% ± 10.5% (range, − 36.0% to − 0.2%). The change in PTV coverage was negligibly small: changes in D_99%_, D_95%_, and D_90%_ were − 0.7% ± 1.2%, − 0.4% ± 1.0%, and − 0.3% ± 0.9%, respectively.

**Table 1:** Mandible dose with and without consideration of lead block attenuation

	SRB_w_ [Gy]	SRB_LB_ [Gy]	Difference [Gy]	*p *value
D_1 cc_	30.3 ± 8.7 (16.7 to 47.7)	27.8 ± 9.6 (13.0 to 47.6)	− 2.5 ± 2.8 (− 10.4 to 0.0)	< 0.001
D_2 cc_	25.8 ± 8.0 (14.1 to 40.9)	23.4 ± 8.4 (10.9 to 40.8)	− 2.4 ± 2.3 (− 8.6 to − 0.1)	< 0.001
D_5 cc_	19.3 ± 6.8 (10.5 to 33.0)	17.4 ± 6.7 (8.9 to 30.2)	− 1.9 ± 1.6 (− 6.1 to 0.0)	< 0.001

Mean ± standard deviation (range) is shown

*D*_*x cc*_ dose delivered to × cm^3^, *SRB*_*w*_ calculation with the ShioRIS Brachy in water, *SRB*_*LB*_ calculation with the SRB with consideration of the lead block attenuation

### Optimization of the treatment plans

**Fig. 7:**
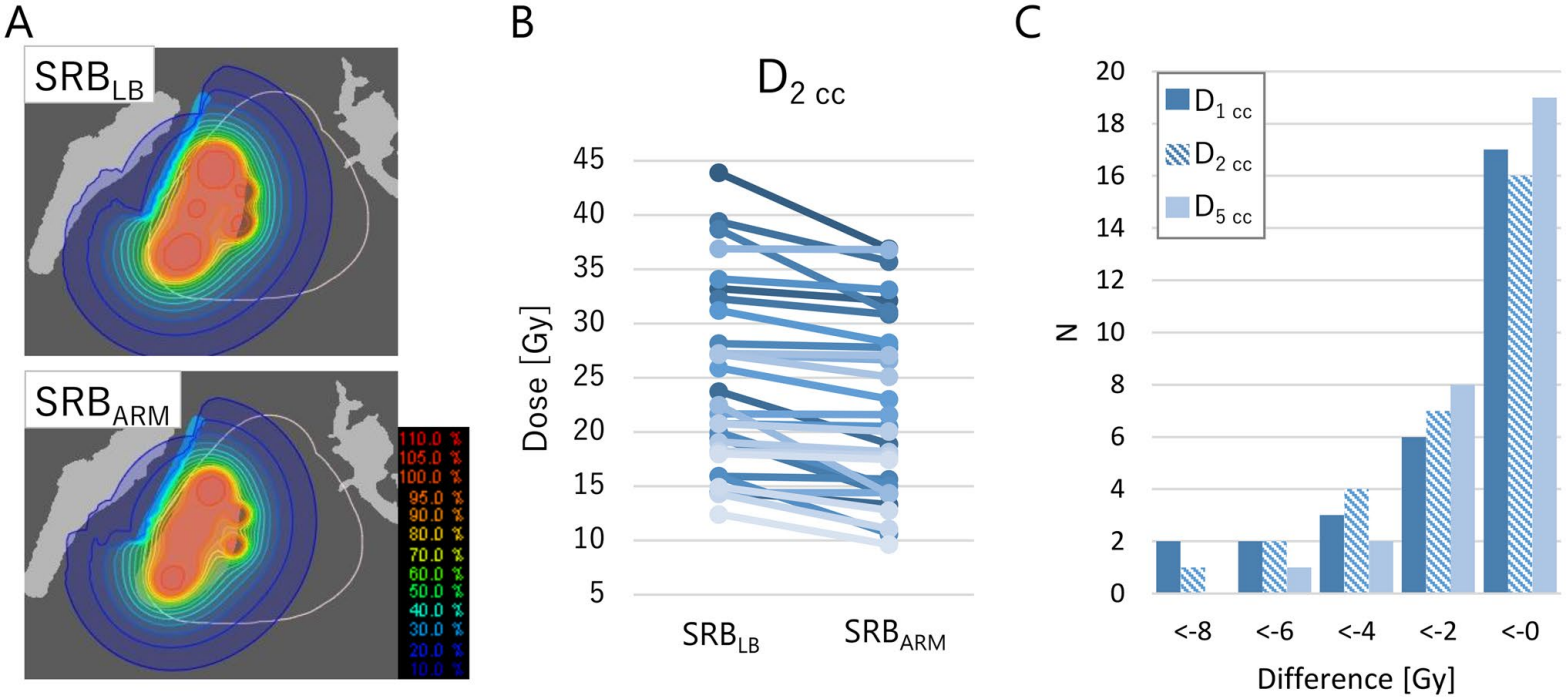
**A** Mandible structure and dose distribution of an example case calculated with ShioRIS Brachy (SRB). *SRB*_*LB*_ calculation with the SRB with consideration of the lead block attenuation, *SRB*_*ARM*_ SRB_LB_ with attraction–repulsion model optimization. **B** Dose delivered to 2 cm^3^ (D_2 cc_) and to 5 cm^3^ (D_5 cc_) of mandible calculated with SRB_LB_ and SRB_ARM_. **C** Number of cases plotted against the differences of the D_1 cc_, D_2 cc_, and D_5 cc_ between SRB_LB_ and SRB_ARM_

Figure 7A shows the example dose distribution of SRB_LB_ (top panel) and SRB_ARM_ (bottom panel). The difference in D_2 cc_ between SRB_LB_ and SRB_ARM_ is shown in Fig. 7B. A decrease in the parameters was observed in some, but not all cases. Differences in D_1 cc_, D_2 cc_, and D_5 cc_ between SRB_LB_ and SRB_ARM_ are shown in Fig. 7C and Table 2. D_2 cc_ decreased by an average of 2.4 Gy. The relative difference in D_2 cc_ was − 10.5% ± 10.6% (range, − 36.4% to 0.0%). Changes in PTV D_99%_ and D_90%_ were − 1.2% ± 2.8% and 0.7% ± 2.0%, respectively.

**Table 2:** Mandible dose with and without ARM optimization

	SRB_LB_ [Gy]	SRB_ARM_ [Gy]	Difference [Gy]	*p *value
D_1 cc_	28.6 ± 9.4 (14.7 to 49.4)	26.1 ± 9.5 (11.3 to 43.0)	− 2.5 ± 2.8 (− 9.7 to 0.0)	< 0.001
D_2 cc_	24.4 ± 8.6 (12.4 to 43.9)	22.0 ± 8.4 (9.6 to 36.9)	− 2.4 ± 2.4 (− 8.2 to 0.0)	< 0.001
D_5 cc_	18.3 ± 7.3 (9.7 to 35.0)	16.4 ± 6.7 (7.8 to 29.5)	− 2.0 ± 1.8 (− 6.8 to 0.0)	< 0.001

Mean ± standard deviation (range) is shown

*D*_*x cc*_ dose delivered to × cm^3^, *SRB*_*LB*_ calculation with the ShioRIS Brachy with consideration of the lead block attenuation, *SRB*_*ARM*_ SRB_LB_ with attraction–repulsion model optimization

## Discussion

In this study, we developed in-house software that can calculate the dose distribution of the ISBT for tongue cancer, considering the attenuation caused by LB inserted in the spacer. We also developed an inverse planning technique that considers the attenuation caused by the LB. As a result, D_2 cc_ in the mandible was reduced by an average of 2.4 Gy with LB, and further dose reduction of the same magnitude was possible with optimization.

García-Consuegra et al. reported DVH constraints for cases of postoperative HDR ISBT in the head and neck and reported a 20-fold increased risk of osteonecrosis of the jaw in cases with D_2 cc_ > 61 Gy [18]. Because this 61 Gy is the physical dose based on a combination of external beam radiotherapy and brachytherapy, this value is not comparable to the present study. The prescribed dose at our institution was 54 Gy/9 fractions. If the equivalent dose in 2 Gy fractions (EQD2) is limited to < 61 Gy, the total physical dose should be less than 40.6 Gy (α/β = 3 Gy). As shown in Fig. 6A, D2_cc_ exceeded 40.6 Gy in only one of the 30 cases, indicating that the distance between the applicator and mandible with the spacer sufficiently reduced the mandibular dose. In addition, the shielding effect was significantly improved by placing the LB inside the space. Therefore, a further reduction of the risk of osteonecrosis of the jaw is expected. However, in the one case whose D_2 cc_ exceeded 40.6 Gy, the decrease in D_2 cc_ by the LB was not observed, indicating the importance of inserting the LB in the proper location.

Akiyama et al. recently evaluated the effect of lead block dose attenuation using a commercial TPS’s advanced collapsed cone dose calculation algorithm. They reported that D_0.1cc_ of alveolar bone was attenuated by approximately half [12]. Compared to their report, the effect of the LB of the present study was modest. They reported that the median thickness of the lead block was 4.2 mm (range, 2.9–4.4 mm), whereas the median thickness of the lead-containing spacer in the present study was 7.2 mm (range, 2.8–14.9 mm). With the thicker spacer, sufficient space was created between the mandible and the PTV, so that the γ ray decayed according to the inverse square law of distance, and the LB absorbed the dose in a complementary manner. Similarly, Obinata et al. reported that 18.2% of patients with a spacer thickness of less than 10 mm developed osteonecrosis in a clinical study using low-dose-rate ISBT, whereas no osteonecrosis occurred when the spacer thickness was greater than 10 mm [19].

The limitation of this study is that the heterogeneity correction was not applied to tissues other than LB. However, Peppen et al. compared the AAPM TG-43-based calculation with Monte Carlo dose calculations and reported that the dose difference in head and neck ISBT was within 2% [20]. Most papers evaluating the correlation between mandibular dose and jaw osteonecrosis in head and neck ISBT have used TG-43-based dose calculations. Therefore, the results of the present study can be compared with previous reports.

To reduce the mandible dose, lead block insertion to proper position is very important. For cases with improper lead block insertion, it would be difficult to adequately reduce the mandible dose by ARM alone. For cases with mandible partially shielded by the lead block, however, the ARM would have potential advantages in terms of improving the treatment plan compared with manual planning because of the complicity of planning and calculation.
